# UBE1a Suppresses Herpes Simplex Virus-1 Replication

**DOI:** 10.3390/v12121391

**Published:** 2020-12-04

**Authors:** Marina Ikeda, Akihiro Ito, Yuichi Sekine, Masahiro Fujimuro

**Affiliations:** 1 Department of Cell Biology, Kyoto Pharmaceutical University, Kyoto 607-8412, Japan; kd17002@ms.kyoto-phu.ac.jp (M.I.); sekine@mb.kyoto-phu.ac.jp (Y.S.); 2 Laboratory of Cell Signaling, School of Life Sciences, Tokyo University of Pharmacy and Life Sciences, Tokyo 192-0392, Japan; aito@toyaku.ac.jp

**Keywords:** herpes simplex viruses, herpesvirus, major capsid protein, lytic replication, ubiquitination, ubiquitin activating enzyme, E1, UBA1, UBE1

## Abstract

Herpes simplex virus-1 (HSV-1) is the causative agent of cold sores, keratitis, meningitis, and encephalitis. HSV-1-encoded ICP5, the major capsid protein, is essential for capsid assembly during viral replication. Ubiquitination is a post-translational modification that plays a critical role in the regulation of cellular events such as proteasomal degradation, protein trafficking, and the antiviral response and viral events such as the establishment of infection and viral replication. Ub-activating enzyme (E1, also named UBE1) is involved in the first step in the ubiquitination. However, it is still unknown whether UBE1 contributes to viral infection or the cellular antiviral response. Here, we found that UBE1a suppressed HSV-1 replication and contributed to the antiviral response. The UBE1a inhibitor PYR-41 increased HSV-1 production. Immunofluorescence analysis revealed that UBE1a highly expressing cells presented low ICP5 expression, and vice versa. UBE1a inhibition by PYR-41 and shRNA increased ICP5 expression in HSV-1-infected cells. UBE1a reduced and retarded ICP5 protein expression, without affecting transcription of ICP5 mRNA or degradation of ICP5 protein. Additionally, UBE1a interacted with ICP27, and both partially co-localized at the Hsc70 foci/virus-induced chaperone-enriched (VICE) domains. PYR-41 reduced the co-localization of UBE1a and ICP27. Thus, our findings provide insights into the mechanism of UBE1a in the cellular response to viral infection.

## 1. Introduction

Herpes simplex virus-1 (HSV-1) gene expression is controlled in a temporal and sequential manner, in which immediate early (IE), early (E), and late (L) genes are expressed sequentially. The expression of each group of proteins is dependent on the expression of the preceding group [1,2]. Some IE gene products function as triggers for transcriptional activation of E genes associated with viral DNA replication. Expression of structural and capsid proteins encoded in L genes is followed by capsid assembly and viral genome packaging in the nucleus and then nucleocapsid egress. These proteins that cooperate in viral DNA replication, gene expression, and encapsidation occur within globular structures termed as replication compartments in the nucleus [3,4]. Weller et al. recently reported that molecular chaperones such as Hsc70 were relocalized at virus-induced chaperone-enriched (VICE) domains that form adjacent to the viral replication compartment [5,6,7,8,9]. VICE domains are considered to regulate the refolding or degradation of abundant de novo viral proteins in the nucleus, because they contain ubiquitin (Ub) and 26S proteasomes, in addition to Hsc70 [5,8].

In eukaryotes, the Ub proteasome system (UPS) consists of an enzymatic cascade that conjugates Ub onto the target proteins to mark them for degradation by the 26S proteasome. Ubiquitination is a highly regulated process conserved in eukaryotes and involved in many fundamental cellular processes [10,11]. Ub conjugation is catalyzed by cellular Ub machinery, consisting of Ub-activating enzyme (E1), Ub-conjugating enzyme (E2), and E3 Ub ligase (E3) in orchestrated, sequential events [12,13]. Initially, the C-terminus of Ub binds to the active Cys of E1 in an ATP-dependent manner [14]. This process requires an initial ATP-dependent activation of Ub with the release of PP_i_, followed by binding of Ub to the active Cys of E1 through a thioester linkage. Subsequently, activated Ub is transferred to E2. Finally, the E2–Ub complex and the target protein are brought together by E3, which facilitates the transfer of activated Ub from E2 to Lys of the target protein and catalyzes processive polyubiquitin (poly-Ub) chain formation. Poly-Ub chains linked through Lys48 function as a signal for proteasomal degradation, whereas other poly-Ub chains (e.g., Lys63- or Lys28-linked chain) and mono-ubiquitination are involved in proteasome-independent events such as endocytosis, protein trafficking, DNA repair, and signal transduction [12,13,15]. A large number of proteins are the target of ubiquitination, and the variety of target proteins is defined by a combination of about 40 kinds of E2s and over 600 E3s. In contrast to E2 and E3, there are only two types of known E1:UBE1 (also known as UBA1) and UBE1L2 (also known as UBA6). In the formation of the poly-Ub chain, UBE1 is largely responsible for Ub activation, which is the initial step of ubiquitination [10]. Several studies have shown that UBE1 is the major isoform that initiates the process of protein ubiquitination, and full deletion of the *UBA1* gene is lethal [16]. UBE1 itself has two isoforms, UBE1a (1058 amino acids; 117 kDa) and UBE1b (1018 amino acids; 110 kDa) [17]. These isoforms are translated from the first start and second AUG codons, respectively, in the translational region of a single mRNA [18]. The main difference between UBE1a and UBE1b is caused by the addition of a nuclear localization signal on the N-terminal of UBE1a. Thus, UBE1a is localized to the nucleus, and UBE1b is mainly localized to the cytosol [17,19].

Viruses have evolved artful strategies to exploit the processes regulated by ubiquitination to promote successful infection by targeting unwanted host proteins (e.g., p53 and MHC molecules) for degradation or stabilizing wanted proteins by de-ubiquitination [20,21,22]. However, it is unknown whether E1 enzyme can influence viral infection or the antiviral cell response. Thus, we evaluated the effects of up and downregulation of UBE1a on HSV-1 infection and found that UBE1a suppressed HSV-1 replication. In this study, we attempted to reveal the mechanism of UBE1a in the antiviral response.

## 2. Materials and Methods

### 2.1. Agents and Plasmids

First, 4[4-(5-nitro-furan-2-ylmethylene)-3,5-dioxo-pyrazolidin-1-yl]-benzoic acid ethyl ester (PYR-41) (Funakoshi, Tokyo, Japan) was dissolved in dimethyl sulfoxide, and cycloheximide (CHX) (FUJIFILM Wako Pure Chemical Corporation, Tokyo, Japan) was dissolved in ethanol. To construct the 2xS-tagged UBE1a-pCIneo for pulldown assay, UBE1a-coding DNA was amplified by PCR from Ube1/PET21d, which was a gift from Cynthia Wolberger (Addgene plasmid # 34,965; http://n2t.net/addgene:34965; RRID:Addgene_34965) [23], and cloned into 2xS-tagged pCIneo [24]. To construct the 2xS-tagged UBE1a-pEF1a-IRES-Neo for establishment of stable cell line, 2xS-tagged UBE1a DNA was cloned into in pEF1a-IRES-Neo, which was a gift from Thomas Zwaka (Addgene plasmid # 28,019; http://n2t.net/addgene:28019; RRID:Addgene_28019) [25]. pLKO-shUBE1-Tet-On was constructed for inducible short hairpin RNA (shRNA) targeting UBE1. Sense and antisense oligonucleotides encoding shRNA against UBE1, 5′-CCGGCCACTGCCTTCTACCTTGTTTCTCGAGAAACAAGGTAGAAGGCAGTGGTTTTT-3′ and 5′-AATTAAAAACCACTGCCTTCTACCTTGTTTCTCGAGAAACAAGGTAGAAGGCAGTGG-3′ were annealed and cloned into the AgeI and EcoRI sites of pLKO-Tet-On, a tetracycline/doxycycline-inducible lentivirus vector.

### 2.2. Virus Infection and Cytopathic Effect (CPE) Assay

**Figure 1:**
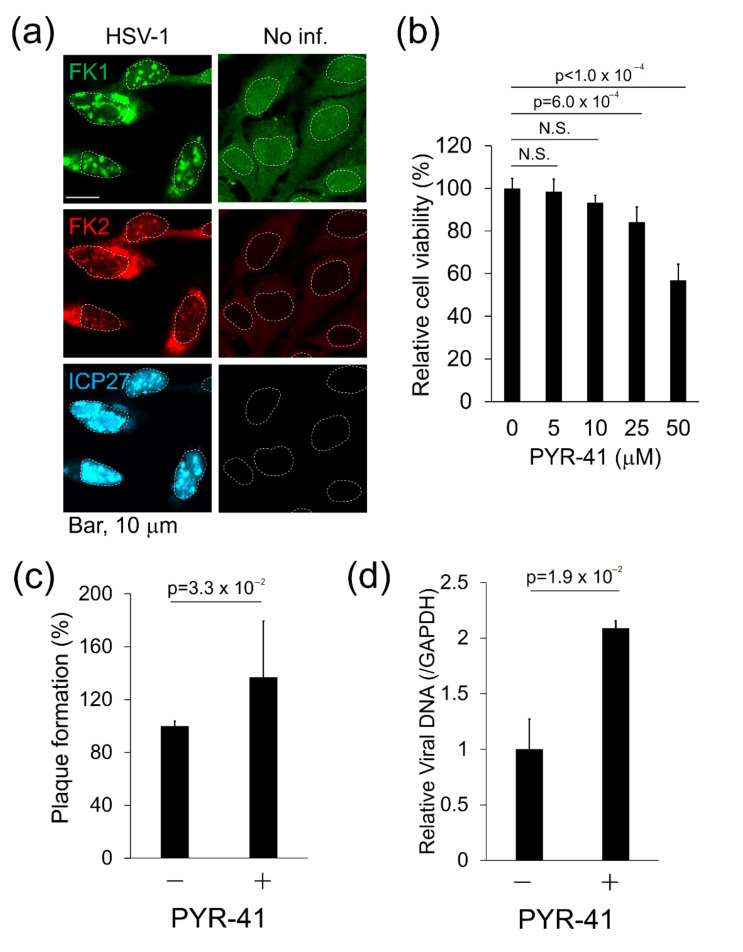
The UBE1 inhibitor PYR-41 increased herpes simplex virus-1 (HSV-1) replication. (**a**) Subcellular localization of Ub-conjugates and UBE1a in HSV-1-infected cells. HeLa cells were infected with HSV-1 at a multiplicity of infection (MOI) of 2 for 60 min. At 18 h post-infection (hpi), cells were fixed and stained with FK1 (green), FK2 (red), and anti-ICP27 (cyan) antibodies. Right and left images show HSV-1 infected and mock-infected cells, respectively. The immediate early (IE) gene product, ICP27, was used as a positive control for HSV infection. The white dotted lines indicate the outline of nuclei. (**b**) Cell viability of PYR-41-treated Vero cells. Vero cells were treated with 0–50 µM PYR-41 and cultured for 48 h. The number of viable cells was evaluated by a cell viability assay. The values obtained from PYR-41-untreated cells were set as 100%. N.S., not significant. (**c**) PYR-41-treatment increased virus production. Vero cells were infected with HSV-1 at 50 plaque-forming units (pfu)/well for 30 min and cultured in media with or without 30 µM PYR-41 for 48 h. The values obtained from PYR-41-untreated cells were set at 100%. Error bars indicate standard deviations from three independent experiments. (**d**) PYR-41 increased viral DNA replication. Vero cells were infected with HSV-1 at an MOI of 0.01 for 30 min and cultured with 25 µM PYR-41 for 24 h. The HSV-1 genome from harvested cells was quantified by real-time PCR and normalized by using total DNA. Values obtained from untreated cells were set as 1.0. Error bars indicate standard deviations from three independent experiments.

The HF strain of herpes simplex virus-1 (HSV-1) was used for infection studies and CPE assays. Vero and HeLa cells were cultivated with Dulbecco’s modified eagle medium supplemented with 10% fetal bovine serum (DMEM–10% FBS). For infection studies, Vero cells seeded in 6-well plates were infected with HSV-1 in 0.2 mL of DMEM for 30 min at a multiplicity of infection (MOI) of 0.01 and subsequently cultured in DMEM–10% FBS. HeLa cells were infected with HSV-1 for 60 min at an MOI of 2 and subsequently cultured in DMEM–10% FBS. For CPE assays, Vero cells were seeded in 12-well tissue culture plates and infected with 50 plaque-forming units of HF (Figure 1d). After a 30 min adsorption, inocula were removed, and cells were cultured in DMEM–10% FBS containing 30 µM PYR-41 and 0.5% methylcellulose. After two days of incubation, maintenance medium was removed. Cells were then stained with 1% crystal violet in 50% methanol, and plaque numbers counted.

### 2.3. Western Blotting (WB), Immunofluorescence Analysis (IFA) and Antibodies

WB and preparation of sample were performed as described previously [26]. IFA was performed as described previously [27]. HeLa and Vero cells for IFA were fixed with 4%-paraformaldehyde (PFA) and permealized in 70% and 100% methanol, on glass slides, followed by incubation with primary antibodies. Primary antibodies used in these experiments included those against UBE1a (#4890) (Cell Signaling Technology, Danvers, MA, USA), β-Actin (sc-69879), UBE1 (sc-53555), ICP0 (sc-53070), ICP5 (sc-56989), ICP8 (sc-53330), ICP27 (sc-69807), Glycoprotein D (gD) (sc-21719), Hsc70 (sc-7298), Glyceraldehyde-3-phosphate dehydrogenase (GAPDH) (M171-3), and S tag (PM021) (MBL, Nagoya, Japan). The FK1 and FK2 antibodies that we established previously [28] were used to detect poly-Ub- and mono-Ub-conjugates. For WB, peroxidase-conjugated goat anti-mouse IgG antibody (115-035-174) (Jackson immune Research, West Grove, MD, USA) and peroxidase-conjugated donkey anti-rabbit IgG antibody (NA934) (GE Healthcare UK Ltd., Buck-inghamshire, UK) were used as these secondary antibody. For IFA, Alexa Fluor-647-conjugated donkey anti-mouse IgG (Invitrogen, Waltham, CA, USA) and Rhodamine-conjugated anti-rabbit IgG antibody (Jackson immune Research) were used as secondary antibodies. For double-staining using two mouse primary antibodies at the same time, Zenon mouse IgG labeling kit (Z25002/Z25007) (Thermo Fisher, Waltham, MA, USA) was used to label a mouse primary antibody with Alexa488 or Alexa594. The nuclei were stained, using Fluoro-KEEPER Antifade Reagent, Non-Hardening Type (Nacalai Tesque, Kyoto, Japan). Immunofluorescent images were obtained via fluorescence microscopy (LSM800; Carl Zeiss, Oberkochen, Germany). Image J software version 1.52a (National Institutes of Health, Bethesda, MD, USA) was used for the analysis of band intensities and the editing of captured images.

### 2.4. Cell Viability Assay

Vero or HeLa cells were seeded in 96-well plates, at 15 × 10^3^ cells/well and 5 × 10^3^ cells/well, in 100 mL of medium, with or without PYR-41, respectively, and then incubated for 48 h. The number of viable cells was calculated with Cell Count Reagent SF (Nacalai Tesque). The optical density at 450 nm of each sample was measured by the spectrophotometer (Tecan M200; Tecan, Kanagawa, Japan).

### 2.5. Quantification of HSV-1 DNA Synthesis

HSV-1 genome in cells was purified and subjected to real-time PCR as described previously [24] to measure intracellular viral DNA replication. HSV-1 genome was purified by using a QIAamp DNA blood mini kit (Qiagen, Germantown, CA, USA), and SYBR green real-time PCR was performed by using HSV-1 specific primers targeting the junction of *UL32* and *UL33* cording flame. The primers for GAPDH were used as an internal control for normalization. The cellular HSV-1 genome was normalized to that of the *GAPDH* gene for quantification. Sequences of primers used were as follows: UL32-UL33-forward 5′-ACTCTTCGCACACCGCG-3′, UL32-UL33-reverse 5′-CGTCTCGCGAGACGTAG-3′, GAPDH-forward 5′-TGCACCACCAACTGCTTAGC-3′, and GAPDH-reverse 5′-GGCATGGACTGTGGTCATGAG-3′.

### 2.6. Establishment of Vero Cells Expressing Tetracycline/Doxycycline-Inducible shRNA

The lentivirus vector for UBE1 knockdown was produced by transfection of pCMV-VSV-G-RSV-Rev, pCAG-HIVgp, and pLKO-shUBE1-Tet-On plasmid into 293T cells. Packaging plasmids (pCMV-VSV-G-RSV-Rev, pCAG-HIVgp) were a kind gift from Dr. Hiroyuki Miyoshi (RIKEN, Wako, JAPAN). Vero cells were transduced with lentivirus as described previously [24], to establish shRNA against UBE1-expressing cells. Dox-inducible shUBE1-Vero cells were cultured in growth medium containing 7.5 μg/mL of puromycin (InvivoGen, San Diego, CA, USA) for selection and maintenance. Vero cells were treated with 2–8 μg/mL of doxycycline (Dox) (LKT Laboratories, St. Paul, MN, USA) for 12 h, to induce shRNA expression against UBE1.

### 2.7. Transfection and Stable Cell Line Generation

HeLa cells seeded at 0.7 × 10^6^ cells pre 6 cm dish were transfected, using the Chen–Okayama calcium phosphate method [29] with 10 µg of plasmid and infected with HSV-1 12 h after transfection. The 2xS tagged UBE1a-pEF1a-IRES-Neo was transfected into HeLa cells by the calcium phosphate method, to establish cell lines stably expressing UBE1a. Transfected cells were selected and maintained in DMEM medium containing 500 µg/mL G418 (Nacalai Tesque).

### 2.8. Reverse Transcription Polymerase Chain Reaction (RT-PCR) and Real-Time-qPCR

Reverse transcription reaction and real-time PCR were performed as described previously [30]. cDNAs, encoding ICP5, ICP27, ICP8 and Actin, were amplified with the following primer pairs: ICP5-forward 5′-GTCGCATCGACGCCTGTTTG-3′, ICP5-reverse 5′-AGAAAGCGCACGAGCGACAG-3′, ICP27-forward 5′-TGCGGCCCTTTCTCCAGT-3′, ICP27-revers 5′-TGCGTGTCTAGGATTTCG-3′, ICP8-forward 5′-GACATTACGTTCACGGCCTTCGAAGCCAG-3′, ICP8-revers 5′-GGCCCGAGTTGGTGCTAAATACCATGGC-3′, Actin-forward 5′-AGAGCTACGAGCTGCCTGAC-3′, and Actin-reverse 5′-AGCACTGTGTTGGCGTACAG-3′. Relative ICP5, ICP27 and ICP8 mRNA expression levels were determined by Actin expression and ΔΔCT threshold cycle (CT) methods.

### 2.9. In Situ Detergent Extraction, DNase, or RNase Treatment

For the detection of detergent-resistant and chromatin-associated nuclear proteins, an in situ extraction method that removes the cytoplasm and nucleosolic proteins was used [8]. This method allows for the detection of the subpopulation of factors that are chromatin-associated and which may otherwise be obscured by the nucleoplasmic pool. HSV-1 infected HeLa cells were extracted on ice for 15 min, using ice-cold modified cytoskeleton (mCSK) buffer (25 mM piperazine-*N*,*N*’-bis (2-ethanesulfonic acid) (PIPES) (pH 6.8), 300 mM sucrose, 1 mM MgCl_2_, 1 mM EDTA, 50 mM NaCl, 8 mM NaF, 1 mM phenylmethylsulfonyl fluoride, and 0.5% Triton X-100). Cells were fixed in 4% PFA, at room temperature, for 15 min, and prepared for IFA as described above. For DNase or RNase treatment, cells were treated with ice-cold mCSK buffer for 10 min and washed twice with PBS. These cells were digested with 100 mg/mL DNase-I (Takara Bio Inc., Shiga, Japan) or RNase-I in PBS containing 5 mM MgCl_2_, respectively. After digestion, cells were washed with ice-cold PBS, fixed in 4% PFA for 15 min, and prepared for IFA as described above.

### 2.10. Pulldown Assays

Cells were lysed with 1.5 mL lysis buffer (10 mM Tris/HCl pH 8.5, 150 mM NaCl, 5% glycerin, 0.1% Nonidet P-40, 1 mM dithiothreitol, and 20 µM phenylmethylsulfonyl fluoride). Cell lysates were incubated with 45 µL S-protein agarose beads, for 1 h, at 4 °C, to pulldown S-tagged UBE1a. Beads were washed with Lysis buffer three times. Clarified beads were resuspended in 70 µL sample buffer with 4% 2-ME.

### 2.11. Statistics

Statistical analysis of Figure 1b and Figure 3a used Tukey′s multiple comparison test. In other figures, a two-tailed Student’s *t*-test was used to indicate the differences between the groups. The *p*-values are shown in each graph.

## 3. Results

### 3.1. UBE1 Inhibitor PYR-41 Enhances HSV-1 Production

First, we used Immunofluorescence analysis (IFA) to investigate the effect of HSV-1 infection on ubiquitination. We previously established that FK1 and FK2 antibodies recognize both mono- and poly-Ub conjugated proteins but not free Ub [28,31]. HeLa cells were infected with HSV-1 at an MOI of 2 for 60 min and were stained at 18 h post-infection (hpi) with FK1 and FK2 (Figure 1a). IFA showed that Ub conjugates were slightly distributed in the nuclei and cytoplasm of both non-infected and infected cells. In contrast, the formation of Ub-conjugates, which seemed to be aggregates, was detected in nuclei after infection. These results suggest that HSV-1 infection affects the ubiquitin–proteasome system (UPS) and increases Ub-conjugates in the host cell. 

In the UPS, UBE1 plays a key role in Ub activation, which is the initial step of polyubiquitination. However, the contribution of E1 enzyme (UBE1) for the establishment of viral infection or the antiviral cell response remains unknown. Accordingly, we investigated the effect of UBE1 inhibition on HSV-1 replication. The UBE1 inhibitor PYR-41 (also known as 4[4-(5-nitro-furan-2-ylmethylene)-3,5-dioxo-pyrazolidin-1-yl]-benzoic acid ethyl ester) selectively suppresses Ub-activating activity of UBE1 [32]. The cytotoxic effect of PYR-41 on Vero cells was tested, and treatment with over 50 µM PYR-41 reduced the viability of Vero cells (Figure 1b). Next, we used the CPE assay to analyze whether PYR-41 affects the production of infectious viruses (Figure 1c). Vero cells were infected with HSV-1 and were subsequently cultured with media in the presence or absence of 30 µM PYR-41. The number of plaques was increased by PYR-41 treatment compared with no treatment. In addition to virus production, we used real-time PCR to evaluate the effect of PYR-41 treatment on virus DNA replication in infected cells (Figure 1d). HSV-1 DNA replication was also increased in PYR-41-treated cells compared with mock cells. Thus, UBE1 inhibition by PYR-41 enhanced both viral DNA replication and viral production. These results suggest that UBE1 activity contributes to the antiviral response. UBE1 has two isoforms, UBE1a and UBE1b, which are localized in the nucleus and cytosol, respectively [16,17]. Because HSV-1 infection induced the formation of Ub-conjugates in the nuclei (Figure 1a), we focused on UBE1a in the next experiments.

### 3.2. Expression Level of UBE1a Is Negatively Correlated with ICP5 Expression

**Figure 2:**
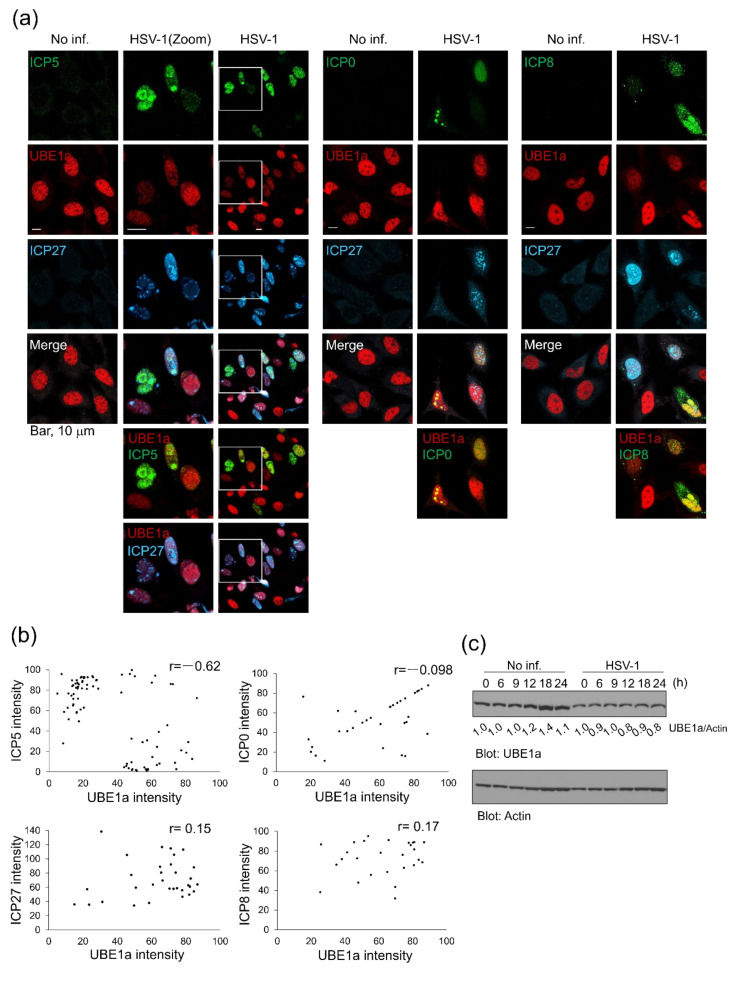
The expression level of UBE1a was a negatively correlated with ICP5 expression. (**a**) HeLa cells were infected with HSV-1 at an MOI of 2 for 60 min and fixed at 18 hpi (hours post-infection). The localization of UBE1a with ICP5 (late (L) gene product), ICP0 (IE gene product), ICP27 (IE gene product), and ICP8 (early (E) gene product) was observed by confocal microscopy. (**b**) The correlations of fluorescence intensities between UBE1a and the nuclear-localizing virus protein, ICP5 (*n* = 97), ICP0 (*n* = 43), ICP27 (*n* = 31), and ICP8 (*n* = 27), were plotted. (**c**) The amount of UBE1a expression did not change either no infection or HSV-1 infected cells. HeLa cells were infected with or without HSV-1 and harvested at 0, 6, 9, 12, 18, and 24 h. Cell lysates were analyzed for UBE1a and actin antibodies. The band intensities of virus proteins were calculated by using ImageJ software and normalized to actin. The values for UBE1a/actin are presented at the bottom of each lane, and the values for non-infected cells are presented as 1.0.

Figure 1c shows that UBE1 inhibition enhanced HSV-1 production, suggesting that UBE1a contributes to the anti-HSV-1 response. To gain insight into the mechanism by which UBE1a suppresses HSV-1 production, the expression patterns of UBE1a and viral proteins were compared. HeLa cells infected with HSV-1 were cultured for 18 h, and expression of UBE1a and lytic gene products were observed by confocal microscopy. UBE1a and HSV-1 IE gene products (ICP0/*RL2* and ICP27/*UL54*), E gene product (ICP8/*UL29*), or L gene product (ICP5/*UL19*) were visualized by IFA (Figure 2a). As a result, HSV-1 infected cells could be divided into two groups: UBE1a highly expressing cells and UBE1a low-expressing cells. ICP27-, ICP0-, and ICP8-positive cells meant infection with HSV-1. The scatterplot in Figure 2b summarizes the correlation between the expression of HSV-1 protein and the expression of UBE1a in HSV-1-infected cells. Interestingly, UBE1a highly expressing cells had a low ICP5 expression, and vice versa. A Pearson product–moment correlation coefficient was computed to assess the relationship between UBE1a expression and viral proteins. This coefficient indicates that UBE1a expression is negatively correlated with ICP5 expression (r = −0.62, *p* < 0.001) (Figure 2b). However, UBE1a expression had no relationship to ICP0- (r = −0.098), ICP27- (r = 0.150), or ICP8- (r = 0.166) expression. Because HSV-1 infection produced UBE1a highly expressing cells and UBE1a low-expressing cells (Figure 2a), these findings suggest the possibility that the expression levels of UBE1a change during HSV-1 lytic replication. We also observed the unequal expression level of UBE1a in uninfected HeLa cells. The previous study showed that the expression levels of UBE1a and UBE1b in HeLa cells are dramatically changed in a cell-cycle-dependent manner [33]. Thus, it can be thought to be reasonable for UBE1a unequal expression in the uninfected cells. However, Western blotting (WB) showed that the total amount of UBE1a changed neither mock cells nor infected cells during lytic replication (Figure 2c).

### 3.3. UBE1a Inhibition Increases ICP5 Expression in HSV-1 Infected Cells

**Figure 3:**
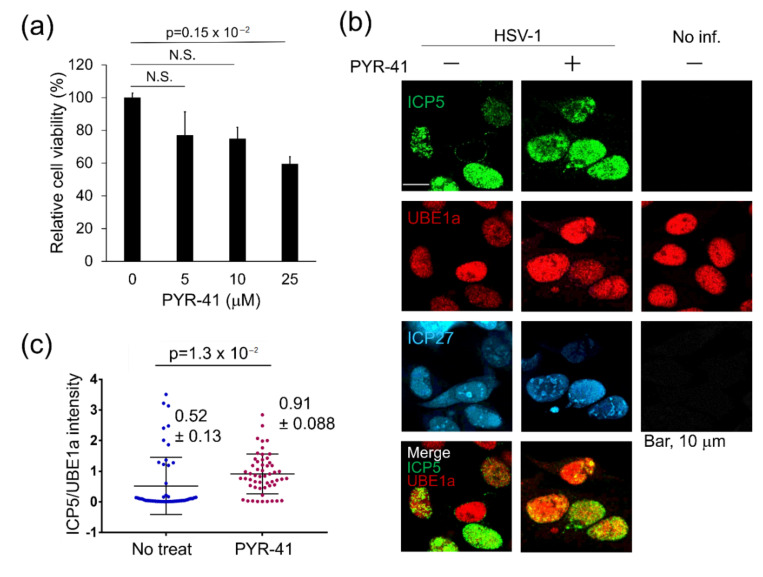
UBE1a inhibition by PYR-41 increased ICP5 expression in HSV-1 infected cells. (**a**) Cell viability of PYR-41 treated HeLa cells. HeLa cells were treated with 0–25 µM PYR-41 and cultured for 48 h. The number of viable cells was evaluated by using a cell viability assay. The values obtained from PYR-41-untreated cells were set as 100%. N.S., not significant. (**b**) PYR-41 increased ICP5 expression in UBE1a highly expressing cells. HeLa cells infected with or without HSV-1 were cultured with 10 µM PYR-41. At 18 hpi, cells were fixed and stained with anti-ICP5 (green), UBE1a (red), and ICP27 (cyan) antibodies. (**c**) The correlation of fluorescence intensity between UBE1a and ICP5 in infected cells in the absence or presence of PYR-41. The means ± standard deviations are shown (*n* = 54 in each experiment). The *y*-axis is ICP5 intensity/UBE1a intensity measured by Image J.

Based on the data of Figure 1 and Figure 2, it can be speculated that UBE1a suppresses HSV-1 production through ICP5 downregulation. Therefore, we investigated whether inhibition of UBE1a influences ICP5 expression in UBE1a highly expressing cells. HeLa cells infected with HSV-1 were cultured with media in the presence or absence of 10 µM PYR-41, which did not influence the viability of HeLa cells (Figure 3a). Subsequently, cells were observed by IFA to analyze the expression of ICP5 and UBE1a (Figure 3b). As expected, UBE1a highly expressing cells expressed ICP5 under treatment with PYR-41. PYR-41 treatment significantly enhanced ICP5 expression in UBE1a-expressing cells, as compared with non-treated UBE1a-expressing cells (Figure 3c). These results suggest that UBE1a suppresses the ICP5 expression through the UBE1a activity (Figure 3c). 

**Figure 4:**
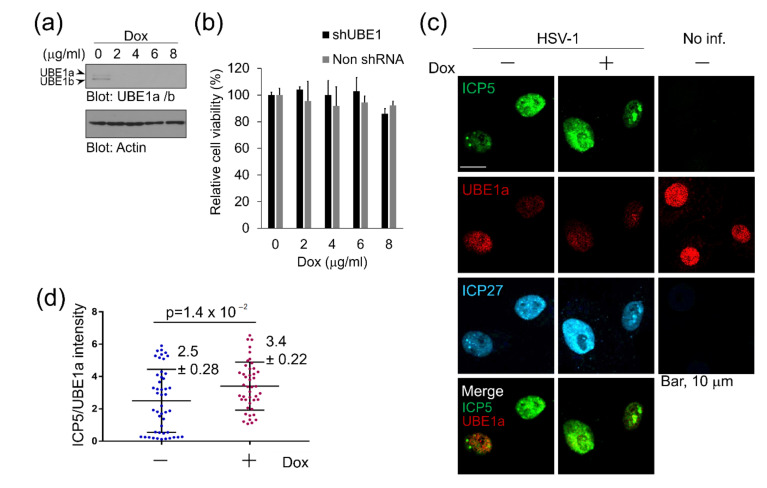
UBE1a knockdown by shRNA increased ICP5 expression in HSV-1 infected cells. (**a**) UBE1-knockdown by Dox-induced shRNA (shUBE1) against UBE1. Western blotting data showed the expression levels of UBE1a and UBE1b. Vero cells were transduced with or without lentivirus encoding Dox-inducible shUBE1, which downregulates both UBE1a and UBE1b. Transduced cells were treated with 0–8 µg/mL Dox for 24 h, and cell lysates were analyzed by Western blotting, using a UBE1 antibody that recognized both UBE1a and UBE1b. (**b**) The cytotoxic effect of Dox on Dox-inducible shUBE1-Vero cells. Cells were treated with 0–8 µg/mL Dox and cultured for 24 h. The number of viable cells was evaluated by a cell viability assay. The values obtained from Dox-untreated cells were set as 100%. (**c**) UBE1 knockdown increased ICP5 expression in HSV-1 infected cells. Vero cells infected with or without HSV-1 were cultured with 6 µg/mL Dox. At 8 hpi, cells were fixed and stained with anti-ICP5 (green), UBE1a (red), and ICP27 (cyan) antibodies. (**d**) The correlation of fluorescence intensity between UBE1a and ICP5 in infected cells after UBE1-knockdown. The means ± standard deviations are shown (*n* = 47, 44 in each experiment). The *y*-axis is ICP5 intensity/UBE1a intensity calculated by using ImageJ.

Furthermore, we investigated the effect of lentivirus-derived shRNA knockdown against UBE1a on ICP5 expression. First, Vero cells were transduced with a lentivirus vector encoding tetracycline/doxycycline (Dox)-inducible shRNA (shUBE1) against UBE1, and stably transduced cells were established by puromycin selection. Our shUBE1 targeted both UBE1a and UBE1b, because UBE1a and UBE1b are known to be translated from the first start AUG codon and the second AUG codon, respectively, in the translational region of a single mRNA [18]. We confirmed UBE1a knockdown by Dox-induced shUBE1 (Figure 4a) and the effect of UBE1a knockdown on the viability of Vero cells (Figure 4b). Expression of shUBE1 by over 2 µg/mL Dox reduced the expression levels of both UBE1a and UBE1b in Vero cells without influencing cell viability (Figure 4a,b). As in Figure 3, UBE1a-downregulated cells by shUBE1 showed significantly enhanced ICP5 expression, compared with non-Dox-treated cells (Figure 4c,d). These results suggest that the activity of UBE1a is involved in the suppression of ICP5 expression.

### 3.4. UBE1a Reduces and Retards ICP5 Protein Expression

**Figure 5:**
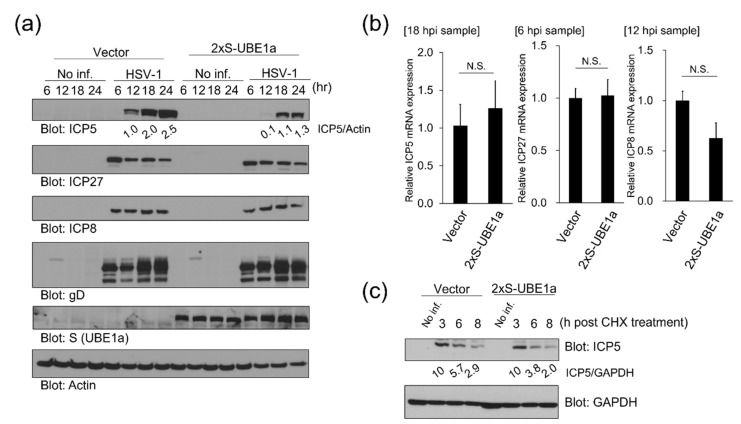
UBE1a overexpression reduced and retarded ICP5 protein expression. (**a**) UBE1a reduced and retarded ICP5 protein expression in HSV-1-infected cells. Polyclonal HeLa cell lines stably transfected with 2xS tagged UBE1a or empty vector were infected with HSV-1, and cells were harvested at 6–24 hpi. Cell lysates were analyzed by Western blotting, using anti-ICP5 (L gene product), ICP27 (IE gene product), ICP8 (E gene product), Glycoprotein D (gD) (L gene product), and S-tag antibodies. ICP5 band intensities were calculated using ImageJ software and normalized to actin. Values of ICP5/actin are presented at the bottom of each lane, and the value of the control vector cell line is presented as 1.0. (**b**) The effects of UBE1a on the mRNA expression of ICP5, ICP27, and ICP8. HeLa cells stably expressing 2xS-UBE1a were infected with HSV-1, and cells were harvested at 6, 12, and 18 hpi. Total RNA extracted from each sample was subjected to RT-qPCR. The expression levels of mRNAs, ICP27 (IE, multifunctional expression regulator), ICP8 (E, major DNA-binding protein), and ICP5 (L, major capsid protein), were normalized by actin mRNA. Expression levels were assessed using 3–4 independent experiments, and error bars indicate ±  standard deviations. N.S., not significant. (**c**) The effects of UBE1a on destabilization of ICP5. Polyclonal HeLa cells stably expressing 2xS-UBE1a infected with HSV-1 were cultured for 18 h, and cycloheximide (CHX) (final concentration, 25 µg/mL) was added into media. Cells were harvested at 3, 6, and 8 h after CHX treatment. The band intensities of virus proteins were calculated using ImageJ software and normalized to GAPDH. The values for ICP5/GAPDH are presented at the bottom of each lane, and the value for 3 hpi cells is presented as 10.

To obtain more direct evidence of UBE1a-mediated suppression of ICP5 expression, we analyzed the influence of UBE1a-overexpression on the expression patterns of HSV-1 lytic protein. HeLa cells stably expressing 2xS-UBE1a were infected with HSV-1, and cells harvested at 6–24 hpi were subjected to WB. No significant changes in the timing of ICP27, ICP8, or Glycoprotein D (gD) expression were observed between non-expressing cells and 2xS-UBE1a over-expressing cells. However, ICP5 expression in S-UBE1a over-expressing cells occurred later than expression in non-expressing cells (Figure 5a). Moreover, the expression level of ICP5 in S-UBE1a over-expressing cells was less than in non-expressing cells. To determine whether a decrease in ICP5 expression in UBE1a overexpressing cells was due to a decrease in ICP5 mRNA, we examined the transcription of viral mRNAs. However, we found no change in mRNA expression of ICP5, ICP27, or ICP8 between 2xS-UBE1a-transfected cells and vector-transfected cells (Figure 5b). Next, to evaluate the effect of UBE1a-overexpression on the destabilization of ICP5, a cycloheximide (CHX) chase analysis was performed. HeLa cells stably expressing 2xS-UBE1a were infected with HSV-1 and cultured for 13 h. Cells were treated with 25 µg/mL CHX and harvested at 3, 6, and 8 h after CHX addition. CHX was used for inhibition of de novo protein synthesis. No significant change was observed in the destabilization of ICP5 between 2xS-UBE1a over-expressing cells and non-expressing cells (Figure 5c). These results suggest that UBE1a reduces and retards ICP5 protein expression without affecting either transcription of ICP5 mRNA or degradation of ICP5 protein.

### 3.5. UBE1a and ICP27 Partially Co-Localizes at the Hsc70 foci/VICE Domains

**Figure 6:**
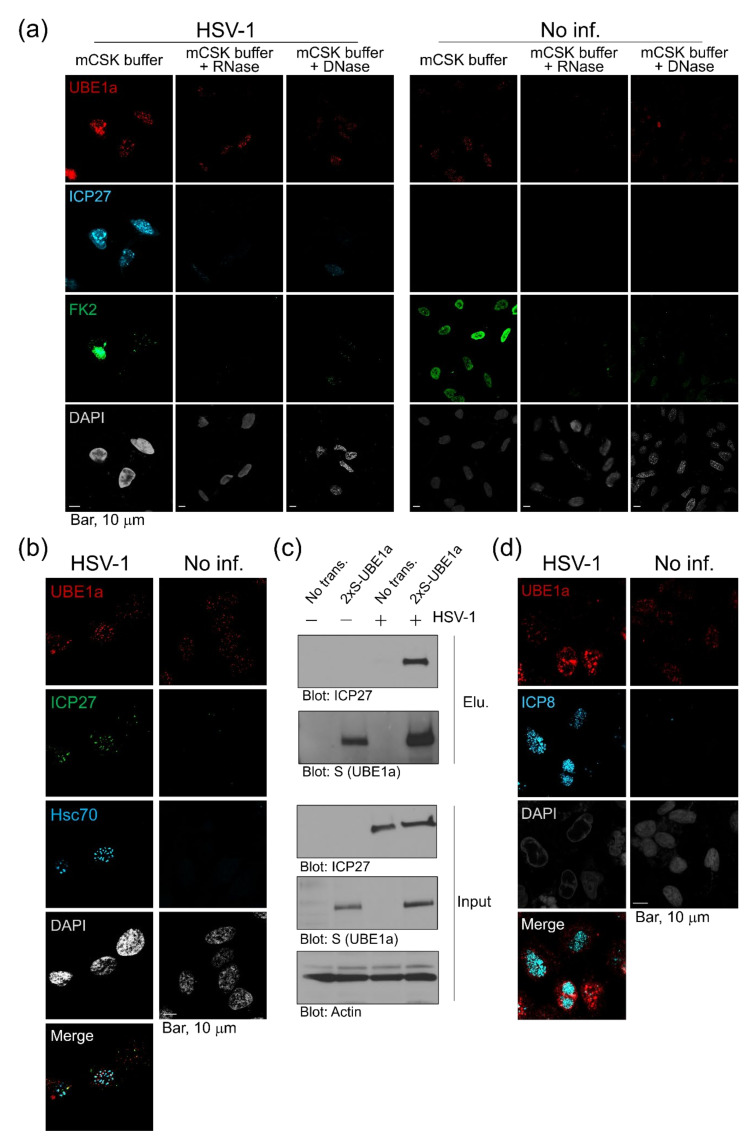
UBE1a interacted with ICP27, and both proteins partially co-localized at the Hsc70 foci/virus-induced chaperone-enriched (VICE) domains. (**a**) UBE1a, ICP27, and Ub-conjugates partially co-localized via DNA and RNA. HSV-1-infected HeLa cells were cultured for 18 h and were extracted with ice-cold modified cytoskeleton (mCSK) buffer. Cells were treated with DNase-I or RNase-I and fixed with 4% paraformaldehyde (PFA). Samples were stained with triple antibodies, FK2 (green), anti-UBE1a (red), and ICP27 (cyan). (**b**) UBE1a and ICP27 were partially co-localized at Hsc70 foci/VICE domains in infected cells. Infected cells at 18 hpi were extracted with ice-cold mCSK buffer and fixed with PFA. Cells were stained with anti-ICP27 (green), UBE1a (red), and Hsc70 (cyan) antibodies. (**c**) UBE1a interacted with ICP27 in HSV-1 infected cells. HeLa cells transfected with 2xS-UBE1a were infected with HSV-1 at an MOI of 5. Cell lysates prepared at 12 hpi were subjected to pulldown assay, using S-protein beads. The precipitated UBE1a were analyzed by blotting with an anti-ICP27 antibody. “Input” means total cell lysates, and 2% cell lysates used for pulldown were applied on SDS-PAGE. (**d**) UBE1a did not localize at the replication compartment in infected cells. Infected cells at 18 hpi were extracted and fixed in 4% PFA. Cells were stained with anti-UBE1a (red) and ICP8 (cyan) antibodies.

**Figure 7:**
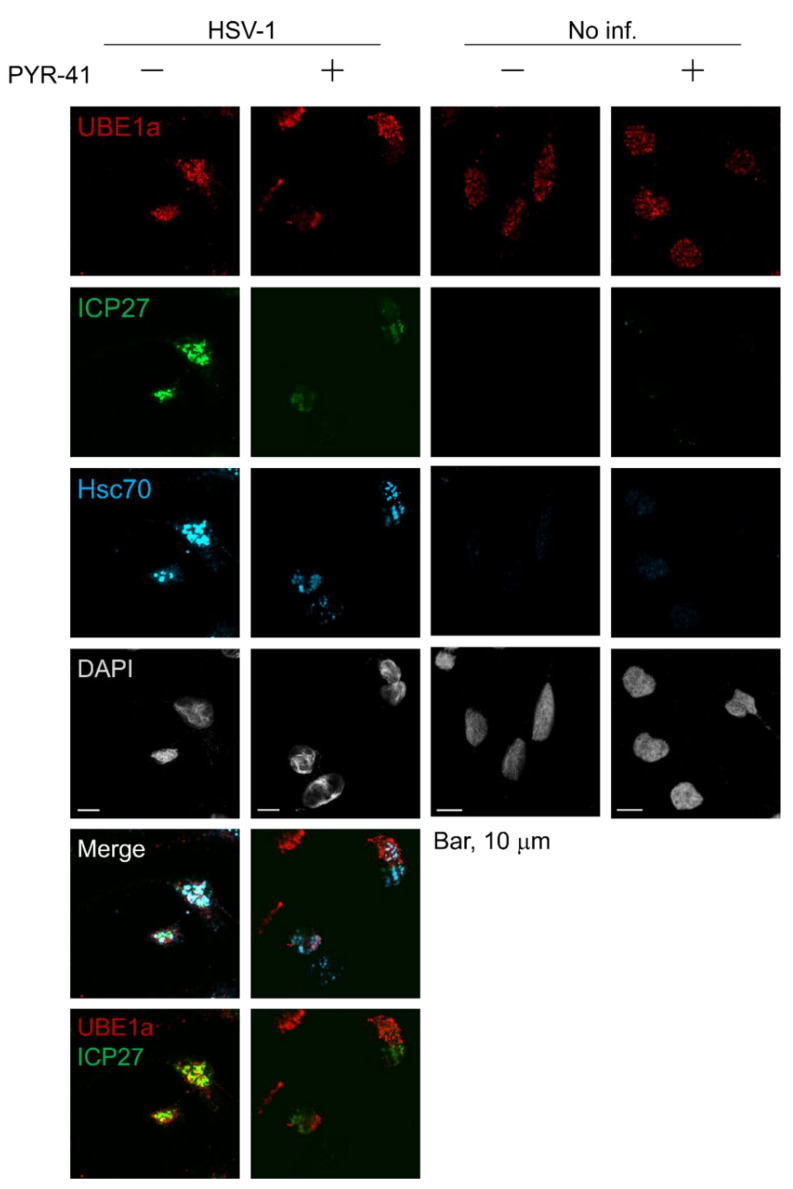
UBE1a inhibitor, PYR-41, decreased the co-localization of UBE1a and ICP27 at the Hsc70 foci/VICE domains in infected cells. HSV-1-infected HeLa cells were cultured for 18 h with or without 10 µM PYR-41 and were extracted with ice-cold mCSK buffer. Cells were fixed in with 4% PFA and stained with anti-ICP27 (green), UBE1a (red), and Hsc70 (cyan) antibodies.

Figure 5 suggests that UBE1a downregulates and retards ICP5 expression by an unknown mechanism. IFA data showed that ICP27 and UBE1a were partially co-localized in nuclei (Figure 2, Figure 3 and Figure 4). ICP27 is known to contribute to the translation of viral mRNAs encoding L gene products (including ICP5 mRNA) by regulating mRNA export from the nucleus into the cytoplasm [34,35]. These findings suggest the possibility that UBE1a downregulates ICP5 expression through interaction with ICP27. To address this point, virus-infected cells were treated by in situ detergent extraction, and detergent-resistant and chromatin-associated nuclear protein clusters were observed by confocal microscopy (Figure 6). The in situ extraction method removes the cytoplasm and nucleosolic proteins from cells. When infected cells are treated with this method, the detergent-resistant and chromatin-associated nuclear protein clusters remain as residual materials. This residue has been reported to contain heat-shock cognate 70 (Hsc70) foci, virus-induced chaperone-enriched (VICE) domains, the viral replication compartment, viral RNA and DNA, ICP8, and FK2-positive Ub-conjugates [5,7,8,9,36,37]. ICP27 is essential for the formation of the Hsc70 foci/VICE domain, at which Hsc70 is localized. VICE domains are thought to be similar to Hsc70 foci and are the cellular location of nuclear protein quality control by proteasomal degradation of misfolded and ubiquitinated proteins [5,7,8,9]. Our data showed that UBE1a, ICP27- and FK2-positive Ub-conjugates were harbored within the nuclear protein clusters of HSV-1 infected cell nuclei (Figure 6a), and they were partially co-localized at the Hsc70 foci/VICE domains, which is supported by data showing the co-localization of ICP27 and Hsc70 (Figure 6b). Further, the interaction of UBE1a and ICP27 was examined by pulldown assay (Figure 6c). HeLa cells transfected with 2xS-UBE1a were infected with HSV-1 and harvested at 12 hpi. The pulldown assay showed the interaction of UBE1a and ICP27 in the HSV-1 infected cells. These results also suggest that UBE1a interacts and co-localizes with ICP27 at the Hsc70 foci/VICE domains. A signal for ICP5 was not detected (data not shown). Additionally, the signals for ICP27 and Ub-conjugates were abolished by DNase-I and RNase-I treatment. Some signals for UBE1a remained even after DNase-I and RNase-I treatments. These results suggest that UBE1a, ICP27, and Ub-conjugates associate with the DNA and RNA of nuclear protein clusters. However, we found that UBE1a and DNA-binding protein ICP8 were localized at the viral replication compartment [9]. UBE1a did not co-localize with ICP8 (Figure 6d), indicating that UBE1a was not localized at the viral replication compartment. Finally, we examined whether UBE1a inhibitor, PYR-41, affected the co-localization of UBE1a and ICP27 at the Hsc70 foci/VICE domains (Figure 7). Interestingly, PYR-41 reduced the co-localization of UBE1a and ICP27 at the Hsc70 foci/VICE domains, indicating that the UBE1a activity is required for the co-localization of UBE1a and ICP27.

## 4. Discussion

We showed an increase in HSV-1 production by UBE1 inhibitor (PYR-41) and a negative correlation between UBE1a expression and ICP5 expression. UBE1a downregulated and retarded ICP5 expression. Furthermore, UBE1a interacted with ICP27, and both proteins partially co-localized at Hsc70 foci/VICE domains. Yang et al. produced the UBE1 inhibitor PYR-41 and reported that it inhibits the formation of a thioester linkage between the active Cys residue of E1 and the C-terminus of Ub [32]. Thus, PYR-41 directly acts on the active Cys residue of UBE1. In this study, PYR-41 eliminated the negative correlation between UBE1a and ICP5 (Figure 3b,c) and induced the production of infectious viruses and replication of the virus genome (Figure 1c,d). In HSV-1 lytic replication, transcriptional products can be divided into three groups: immediate early gene (IE), early gene (E), and late gene (L). The L genes can be further divided into two classes: γ1 (leaky-L genes) occurs at low levels prior to HSV-1 DNA replication, and γ2 (true-L genes) requires IE and E gene products, as well as viral DNA replication for efficient expression [1]. ICP5 belongs to γ1, and ICP5 expression is important for viral replication and viral capsid assembly [38,39]. Based on these previous reports, we think that the increase in viral DNA replication and viral production by PYR-41 is due to the PYR-41-mediated elimination of UBE1a-induced ICP5 downregulation.

This mechanism, by which UBE1a suppresses ICP5 expression, was ascribed to neither inhibition of ICP5 mRNA transcription nor enhancement of ICP5 degradation (Figure 5b,c). Thus, we suggest two possibilities for the inhibition mechanism of ICP5 expression: (i) inhibition of ICP5 mRNA translation by UBE1a and (ii) inhibition of ICP5 mRNA transport by UBE1a. Recently, it has been disclosed that the ubiquitination of ribosomes plays a critical role in the control over the translation of mRNA into protein by ribosomes. For example, ribosome-mediated protein quality control and ribosome activation by mono-ubiquitination have been reported [40,41,42]. However, UBE1a is well-known to be localized in the nucleus, and our IFA data also showed nuclear localization of UBE1a. Therefore, we focused on the possibility of (ii) inhibition of ICP5 mRNA transport by UBE1a. ICP27 is involved in the translation of ICP5 mRNA, as well as other L gene mRNAs by the regulation of mRNA export from the nucleus to the cytoplasm [34,35]. Thus, we attempted to explore the relationship between UBE1a and ICP27. As a result, we found that UBE1a interacted with ICP27 in HSV-1 infected cells (Figure 6c), and UBE1a was partially co-localized with ICP27, Ub-conjugates, and Hsc70 at the VICE domains (Figure 6b). Moreover, PYR-41 canceled the co-localization of UBE1a and ICP27 at the Hsc70 foci/VICE domains (Figure 7), indicating that the UBE1a activity is required for the co-localization of UBE1a and ICP27. It can be speculated that UBE1a activity may be involved in ICP5 downregulation through the interaction of UBE1a and ICP27.

Although Glycoprotein D (gD) belongs to the γ1 gene, as well as ICP5, the expression of gD was not downregulated by the overexpression of UBE1a (Figure 5a). Fontaine-Rodriguez et al. found that ICP27 upregulated ICP5 mRNA but not gD mRNA [34]. The fundamental mechanism was not disclosed; they argued that ICP27 might have the specificity for the target mRNA in the translational regulation, and ICP27-interacting molecules might decide the target mRNA for translational regulation.

Hsc70 foci/VICE domains in virus-infected cell nuclei are thought to be the location of protein quality control in the nucleus, where refolding and ubiquitination-dependent proteasomal degradation target misfolded or abnormal proteins, including viral protein [5,7,8,9]. However, it is unclear whether those abnormal proteins are ubiquitinated and degraded by UPS in the VICE domains. Because UBE1a plays a key role in the initial step of ubiquitination, our results suggest that ubiquitination by UPS occurs in VICE domains but not at the viral replication compartment (Figure 6b–d). To our knowledge, this is the first report to demonstrate the UBE1a-co-localization with ICP27, Ub-conjugates, and Hsc70 at the VICE domains. ICP27-modification by phosphorylation and arginine methylation was reported to regulate the interaction with RNA polymerase II, the complex formation with a VICE domain, and the export of ICP27 protein from the nucleus to the cytoplasm [36,37,43,44]. In addition, MS analysis revealed that ICP27 has a low potential for ubiquitination [45]. Thus, the interaction between UBE1a and ICP27 may prevent the export of ICP5 mRNA. We plan to proceed with a deeper functional analysis.

Ubiquitination is known to be necessary for the innate immune response. When toll-like receptors (TLRs) or herpesvirus entry mediator (HVEM) binds to pathogen-associated molecular patterns (PAMPS) derived from HSV (e.g., gD), these interactions induce the activation of NF-kB and IRF signaling [46,47]. In NF-kB signaling, TRAFs, NEMO/IKKγ, and IkBa are modified with poly-Ub in order to activate NF-kB signaling [48]. Thus, ubiquitination by UPS is necessary for the innate immune response. However, we believe that HSV-1 also exploits the UPS, including UBE1a, to promote viral survival and replication. The UPS is known to be utilized by viruses to achieve virus infection, replication, anti-apoptosis, and evasion from immunity [49]. Viruses have evolved artful strategies to exploit the processes regulated by ubiquitination to promote successful infection by targeting unwanted host proteins for proteasomal degradation or stabilization of wanted proteins by de-ubiquitination. In fact, HSV-1 ICP0 functions as an E3 Ub ligase and mediates the poly-ubiquitination of cdc34 [50], p53 [21], PML, and Sp100 [51] for degradation by the 26S proteasome. Moreover, to enhance its own stabilization, ICP0 interacts with a deubiquitinating enzyme to disassemble poly-Ub chains from poly-ubiquitinated ICP0 [52]. It has also been recently shown that ubiquitination was required for invasion of the nervous system by HSV-1 [45]. Thus, the UPS is employed by both the human immune system and viruses. 

We demonstrate that the UBE1 inhibitor PYR-41 enhanced HSV-1 production, and UBE1a expression was a negatively correlated with ICP5 expression. UBE1a inhibition by PYR-41 and shRNA increased ICP5 expression in HSV-1 infected cells. Furthermore, UBE1a reduced and retarded ICP5 protein expression. UBE1a was partially co-localized with ICP27 and Ub-conjugates at Hsc70 foci/VICE domains. These findings shed light on the mechanism by which UBE1a contributes to the antiviral response in HSV-1-infected cells. Further study to understand the function of UBE1a might lead to the development of novel therapeutic options for HSV-1-related diseases.
